# Nosocomial Transmission of *Plasmodium falciparum* Malaria, Spain, 2024

**DOI:** 10.3201/eid3106.241932

**Published:** 2025-06

**Authors:** Manuel F. Liroa Romero, Maite Ruiz Pérez de Pipaón, Maria D. Navarro Amuedo, Jose M. Rubio Muñoz, Jose M. Jiménez-Hoyuela, Jose M. Cisneros

**Affiliations:** University of Seville, Seville, Spain (M.F. Liroa Romero, M. Ruiz Pérez de Pipaón, M.D. Navarro Amuedo, J.M. Jiménez-Hoyuela, J.M. Cisneros); Hospital Universitario Virgen del Rocío/IBis, Seville (M.F. Liroa Romero, M. Ruiz Pérez de Pipaón, M.D. Navarro Amuedo, J.M. Cisneros); CIBERINFEC, Instituto de Salud Carlos III, Madrid, Spain (J.M. Rubio Muñoz, J.M. Cisneros)

**Keywords:** malaria, Plasmodium falciparum, vector-borne infections, acquired malaria, parenteral transmission, parasites, Spain

## Abstract

We report nosocomial *Plasmodium falciparum* malaria in Spain, which was confirmed microbiologically and genomically. Transmission occurred through insufficiently disinfected reusable syringe lead shielding during thyroid scintigraphy. Genomic analysis showed high similarity between isolates from index and source cases. Strict biosafety measures are needed in healthcare settings to prevent malaria transmission.

Malaria is an infectious disease caused by *Plasmodium* protozoa and is primarily transmitted to humans through the bite of an *Anopheles* mosquito (*1*). Countries without malaria report cases of infection through blood product transfusions (1 case/4 million inhabitants) (*2*) and solid organ transplants (1 case/1 million inhabitants) (*3*). Cases were also reported for which transmission mechanism was not established and a parenteral route was suspected (0.006 cases/1 million inhabitants) (*4*).

In 2022, a total of 6,131 cases of malaria were confirmed in Europe. Fourteen autochthonous cases were caused by *P. falciparum*: 9 cases related to airports, 3 cryptogenic cases (epidemiologic investigations failed to identify an apparent mode of acquisition), and 2 cases acquired in a hospital in Spain (*5*).

In Spain, autochthonous malaria was eradicated in 1964 (*4*). Since then, *P. vivax* malaria was found in 2 autochthonous cases (*6*) and was explained by the presence the *P. vivax* vector *An. atroparvus* mosquito in Spain (*6*). Conversely, *P. falciparum* vectors *An. algeriensis* and *An. plumbeus* mosquitoes are not found in Spain (*7*). In 2024, the annual number of imported malaria cases in Spain was 600 (*4*,*8*); 2 cases of airport transmission and 5 cases of nosocomial acquisition also occurred (*6*). Of the 5 nosocomial acquisition cases, 1 case was linked to a blood product transfusion (*6*), 1 case was linked to organ transplantation (*6*), and 3 cases had no identified nosocomial transmission mechanism (*6*). None of the 5 nosocomial malaria cases had parasite identification in the source patient, and thus, transmission was not confirmed through genomic comparison (*4*). We describe a case of nosocomial malaria acquired in Spain in 2024, with microbiological and genomic confirmation and transmission mechanism identification. 

A 60-year-old woman from Gilena, southern Spain, who had arterial hypertension and was under study for a thyroid nodule sought care at an emergency department with fever (38°C), general malaise, night sweats, and arthralgia lasting 5 days. She had thrombocytopenia (47,000 platelets/µL) and elevated total bilirubin level (2.18 mg/dL, reference range 0.3–1.2 mg/dL). Peripheral blood smear showed abundant erythrocytes infected with *Plasmodium* spp. PCR in blood and thick smear confirmed *P. falciparum* infection with blood parasitemia index of 7%. Intravenous artesunate treatment was initiated, followed by combination oral dihydroartemisinin/piperaquine treatment for 3 days, which resulted in rapid recovery.

We initiated an exhaustive epidemiologic investigation after diagnosing presumably autochthonous *P. falciparum* malaria. The patient confirmed she had never traveled outside Spain, visited airports, been hospitalized, or received blood transfusions or organ transplants. However, 15 days before fever onset, she underwent thyroid scintigraphy with radioactive iodine. The patient who had been tested before her was from Equatorial Guinea; he was asymptomatic, afebrile, had no signs of infection, and had not traveled to his home country in >2 years. However, he reported a history of malaria in childhood. PCR and thick blood smear testing was conducted and identified asymptomatic *P. falciparum* infection with low-grade parasitemia. Genetic analysis comparing *P. falciparum* isolates from both patients, focusing on *PfMSP-1* and *PfMSP-2* (merozoite surface proteins), showed substantial genetic similarity (Figure 1). Of the remaining patients who underwent scintigraphy the same day, none had fever or infection signs within 30 days of the procedure.

**Figure 1:**
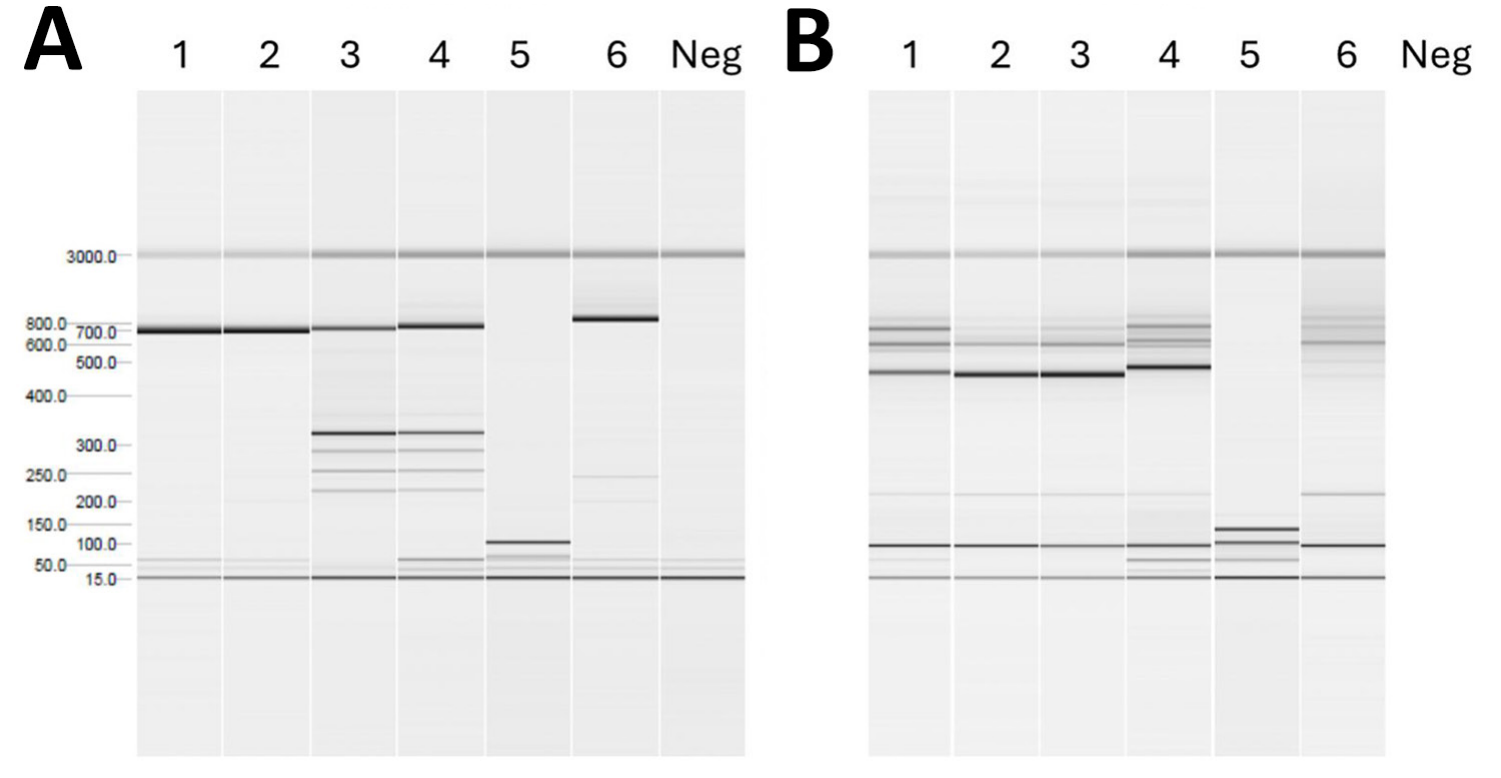
Genotyping study of *Plasmodium falciparum* isolated from 2 patients involved in nosocomial transmission of *P. falciparum* malaria, Spain, 2024. The genes analyzed were *PfMSP-1* and *PfMSP-2*. Results for genotypes FC27 (A) and IC (B) for the MSP-2 families are shown. The findings indicate that the fragments detected in the index case are also present in the source case. MSP, merozoite surface protein; neg, negative.

We reviewed the scintigraphy procedure and confirmed that the syringe was discarded after intravenous administration of radioactive iodine. A lead protector shielded the syringe and needle (Figure 2). Single-dose vials had traceability labels. For thyroid scintigraphy, intravenous administration of the radiopharmaceutical is required. Although blood aspiration is generally avoided, minimal aspiration may occur during venous access. Slight blood aspiration during venous access is the most probable explanation of the nosocomial transmission (https://youtu.be/2OW9g2tiBjc). After administration, the syringe and needle are discarded as radioactive waste, and lead shields are cleaned with 70% isopropyl alcohol and immersed in peroxide-based disinfectants for reuse. Contamination of the new syringe with blood from the previous patient by placing it in the inadequately cleaned sheath was the likely mechanism of *P. falciparum* transmission. However, the lead shield was also reused, after cleaning with antiseptic solution.

**Figure 2:**
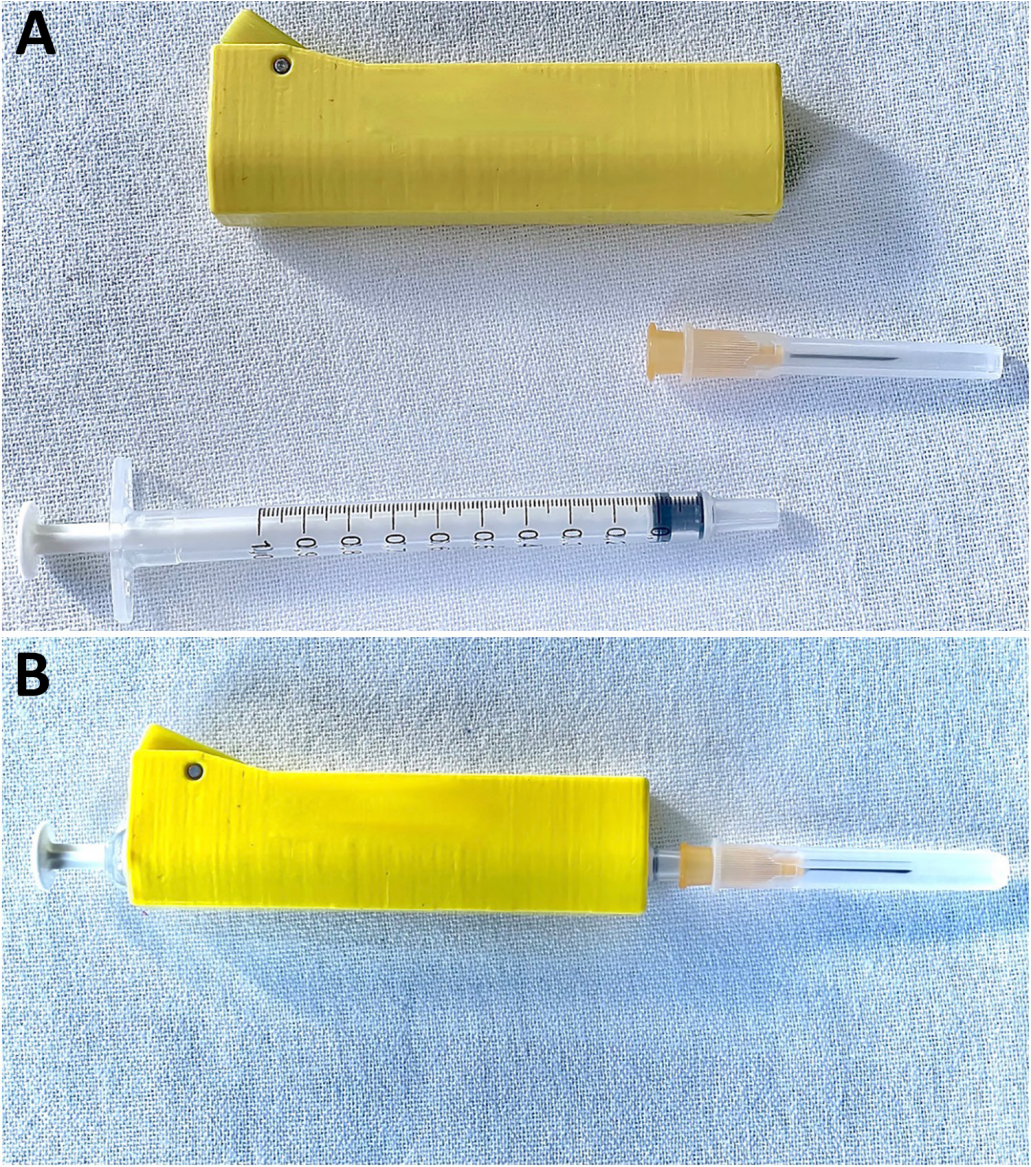
Syringe covered with a lead shield used during nosocomial transmission of *Plasmodium falciparum* Malaria, Spain, 2024. A) Yellow lead shield with removable disposable syringe and needle. B) Syringe assembled with the lead shield.

This study clinically and microbiologically confirms a case of *P. falciparum* acquisition in Spain and describes a nosocomial transmission mechanism through intravenous administration of radioactive iodine during thyroid scintigraphy caused by inadequate disinfection of the reusable lead shield. The sequence of events—scintigraphy performed on the source case followed by the index case, identification of *P. falciparum* in blood of both patients, and genetic concordance of the isolates—shows the transmission mechanism. Our findings helped identify and correct a safety issue in the diagnostic procedure. Each lead cover is now used only 1 time per day and then autoclave sterilized before use another day. 

This case underscores the importance of asymptomatic carriers as reservoirs for malaria transmission, which is a well-known issue in endemic regions (*9*,*10*). Asymptomatic carriers should be considered in nonendemic areas because of globalization and increased healthcare interactions. Malaria should be included in the differential diagnosis for patients with fever and unexplained thrombocytopenia in nonendemic countries. Clinicians must recognize the critical need for stringent biosafety measures and safe practices in healthcare settings.
